# Deep Learning System for User Identification Using Sensors on Doorknobs

**DOI:** 10.3390/s24155072

**Published:** 2024-08-05

**Authors:** Jesús Vegas, A. Ravishankar Rao, César Llamas

**Affiliations:** 1 Escuela de Ingeniería Informática, Universidad de Valladolid, Paseo de Belén 15, 47011 Valladolid, Spain; cesar.llamas@uva.es; 2 Gildart Haase School of Computer Sciences and Engineering, Fairleigh Dickinson University, 1000 River Rd. T-MU1-01, Teaneck, NJ 07666-1914, USA; ravirao@fdu.edu

**Keywords:** access control, user identification, IoT, sensors, machine learning

## Abstract

Door access control systems are important to protect the security and integrity of physical spaces. Accuracy and speed are important factors that govern their performance. In this paper, we investigate a novel approach to identify users by measuring patterns of their interactions with a doorknob via an embedded accelerometer and gyroscope and by applying deep-learning-based algorithms to these measurements. Our identification results obtained from 47 users show an accuracy of 90.2%. When the sex of the user is used as an input feature, the accuracy is 89.8% in the case of male individuals and 97.0% in the case of female individuals. We study how the accuracy is affected by the sample duration, finding that is its possible to identify users using a sample of 0.5 s with an accuracy of 68.5%. Our results demonstrate the feasibility of using patterns of motor activity to provide access control, thus extending with it the set of alternatives to be considered for behavioral biometrics.

## 1. Introduction

Access control systems play a pivotal role in modern security paradigms, ensuring that only authorized individuals gain access to secure premises. The accuracy of these systems is an obvious concern, as erroneous decisions in the form of false positives and false negatives can have substantial security implications. Door access control systems are typically built upon both biometric and non-biometric information [1]. More traditional access control systems are based on the interaction of the users with proximity cards/key fobs and keypad/PIN-entry infrastructures. While proximity cards and key fobs are reliable for granting access, they lack biometric attributes and rely solely on possession. Vulnerabilities such as card theft or cloning have been documented as potential security concerns [2].

On the other hand, access control through keypad/PIN entry relies on the accuracy of user input: a factor influenced by user compliance and the secrecy of personal identification numbers (PINs). Adams et al. [3] emphasize the significance of safeguarding PIN secrecy to mitigate the risk of unauthorized access.

Several approaches for biometric-based control access systems utilize information obtained from images, video, sound, and inertial sensors. Face recognition can offer exceptional accuracy when appropriately configured. State-of-the-art deep-learning-based algorithms have significantly elevated the accuracy of face recognition systems [4]. Nevertheless, environmental factors such as variations in lighting conditions and facial alterations due to age or accessories can introduce variability in performance [5]. In recent years, the advent of RGB-D cameras has revolutionized the ability to obtain 3D information in real-time [6]. These cameras combine traditional RGB imaging with depth perception, enabling detailed capture of spatial and color data simultaneously. This integration facilitates various applications, from facial recognition and gesture tracking to augmented reality and robotics, providing a comprehensive and dynamic view of the environment that enhances both user interaction and technological functionality.

Fingerprint recognition remains one of the cornerstones of access control due to its inherent accuracy and uniqueness. Studies have consistently highlighted the stability of fingerprint patterns over time [7]. Here too, environmental factors, such as the presence of dirt or moisture on fingers, can introduce variability in performance [8].

Iris recognition, due its exceptional accuracy, is also used as a base of access control systems. Numerous studies have stated the stability and uniqueness of iris patterns [9]. Moreover, environmental factors produce a minimal impact on iris recognition, making it a robust choice for access control [10]. Nevertheless, their use for door access control is not widespread due to their cost and complexity compared to other control mechanisms.

Voice biometrics, also known as voice recognition or speaker verification, has gained prominence as an advanced biometric identification method and leverages distinctive vocal characteristics to authenticate individuals [11]. The accuracy of voice recognition has been widely studied, taking into account factors such as microphone quality and background noise, highlighting the influence of background noise and voice alterations on accuracy [12].

Behavioral biometrics represents an emerging domain within the field of biometric authentication and focuses on the inherent and unique behavioral patterns demonstrated by individuals during their interactions with digital systems and devices. Unlike conventional biometrics such as fingerprints or facial recognition, which rely on static physiological attributes, behavioral biometrics capitalizes on the dynamic facets of human behavior [13]. These behaviors encompass a wide spectrum of activities, including keystroke dynamics [14], mouse movements [15], touchscreen gestures [16], and even an individual’s gait [17]. Behavioral biometrics presents a non-intrusive and continuous authentication approach that adapts to users’ innate behaviors, rendering it suitable for a wide range of applications, such as bolstering cybersecurity and enhancing user verification protocols [18].

The motivation for transparent identification of users through interactions with objects such as doorknobs is driven by the need for seamless and unobtrusive authentication methods. Unlike common methods that often require active participation from the user, such as entering passwords or presenting biometric data, object-based interactions offer a passive and more natural approach to identity verification. This method leverages the unique patterns of interaction that individuals have with everyday objects, providing a continuous and non-intrusive means of authentication. As highlighted by research, this approach can significantly enhance user experience by integrating authentication into routine activities, thus improving both security and convenience [19].

In this paper, we analyze the accuracy and performance of a novel and completely transparent approach for automatic person identification based on the interactions of a person with a door handle without the mediation of any extra device brought or worn by the user. The main research question we pose in this paper is to ask if patterns of user interactions with a doorknob can be used reliably for access control. Our research hypothesis is that state-of-the-art classification methods such as deep learning can work well for this problem. We used the system described in [20] to collect sensory information and investigated deep learning techniques for user identification. The results we obtained validate the hypothesis that this technique is valid for access control. Thus, we extend the repertoire of techniques to be considered for behavioral biometrics.

This paper is organized as follows: Section 2 describes the state of the art regarding the identification of users based on interactions with a door; Section 3 describes the dataset, the proposed model, and the parameters for each experiment; Section 4 shows the results of different models we proposed for identifying users; Section 5 deals with the discussion of the results obtained; Section 6 summarizes the main findings and conclusions of our research; finally, Section 7 presents the future work.

## 2. Related Works

In recent years, researchers have studied the feasibility of using accelerometer and gyroscope data from door-mounted inertial measurement units (IMUs) for user identification in access control systems. IMUs provide continuous monitoring of motion dynamics, allowing the capture of unique behavioral patterns associated with an individual’s interactions with a door.

Gjoreski et al. [21] describe an approach for recognizing individuals entering a room by analyzing door acceleration using data collected from 12 users. The results indicate that the best-performing methods achieve an accuracy of 89.5% when combining TD-Feature, TF-Signal, and FD-Feature, confirming the feasibility of identifying users based on their interactions with the door.

Fukao et al. in [22] focused on individual recognition in environments with frequent room access and built a prototype system implemented on a lever-handle door. Experiments involving four subjects validated the approach. The results showed an F-measure of 0.90 for entry motions and 0.73 for exit motions, demonstrating the feasibility of using the motion of turning a doorknob for identification.

Rodriguez et al. in [23] present a system for user identification based on the interaction with a door handle. Their system uses a single IMU sensor attached to the door handle to capture the user’s interaction with the door. They used data from four users and applied feature extraction followed by classification using random forests. The average identification accuracy for the combination of three detection technologies was 84.25% (when opening the door) and 83.5% (when closing the door). Among the different detection technologies, the IMU achieved the highest accuracy, with 90.0% for door opening and 94.75% for door closing. The identification accuracy before the door is opened—specifically, when the handle is pressed but the door has not yet moved—was 70.94%.

Ishida et al. in [24] investigated the identification of individuals who place food in a refrigerator by employing pressure sensors, an accelerometer, and a gyroscope affixed to the refrigerator door. This approach utilizes the motions involved in opening and closing the refrigerator door as well as the pressure distribution during gripping of the door handle. Their method achieves a user identification accuracy of 91.9% using a Bayesian network classifier applied to features obtained from a group of four individuals.

Instead of user interactions with a door, Han et al. [25] studied interactions with a set of different objects in a kitchen. Their results showed that it is possible to correctly identify occupants in 96% of the trials without labeled training data, while sensor identification alone yields an accuracy of 74% even with training data. The identification was done using supervised learning techniques and K-means clustering over the features extracted from the information given by accelerometers.

### Deep Learning Architectures for User Identification

Deep learning architectures have emerged as indispensable tools for user identification tasks, leveraging sensor data to extract complex patterns and achieve high levels of accuracy. Among these architectures, convolutional neural networks (CNNs), recurrent neural networks (RNNs), transformers, and their variants stand out for their ability to adapt to diverse identification scenarios encompassing spatial and temporal aspects of user interactions with sensors.

Convolutional neural networks (CNNs) are a class of deep learning architectures designed for processing structured grid-like data such as images [26], time-series data, and spatial data. CNNs have revolutionized various fields, particularly computer vision, due to their ability to automatically learn hierarchical features from raw input data [27].

Recurrent neural networks (RNNs) are a class of neural network architectures specifically designed to handle sequential data due to their ability to maintain a memory of past inputs by passing information from one time step to the next through recurrent connections. This makes RNNs well-suited for tasks such as time-series prediction, language modeling, sentiment analysis, and machine translation [28].

However, standard RNNs suffer from the vanishing gradient problem, which limits their ability to capture long-term dependencies in sequential data. To address this issue, several variants of RNNs have been developed, including long short-term memory networks (LSTMs) and gated recurrent units (GRUs).

LSTMs and GRUs introduce gating mechanisms that control the flow of information through the network, making them better suited for tasks involving longer sequences.

LSTMs excel in tasks requiring memory of past sensor observations, such as activity recognition and user authentication over extended periods [29].

GRUs were introduced as a simplified alternative to LSTMs, making them well-suited for applications with limited computational resources or large-scale datasets [30].

Transformers rely heavily on attention mechanisms, wherein attention is used to calculate the importance of each element in the input sequence relative to all other elements. The key innovation of transformers lies in their ability to perform self-attention in parallel across all elements in the sequence, enabling efficient processing of long-range dependencies without the need for recurrent connections [31].

Transformers offer flexibility and scalability in modeling user interactions with sensors, enabling adaptation to diverse identification scenarios and data modalities [32].

Deep learning architectures, including CNNs, RNNs, transformers, and their variants, offer versatile solutions for user identification tasks across a wide range of sensor-based applications. By combining spatial-, temporal-, and attention-based modeling capabilities, these architectures enable robust and accurate identification, contributing to enhanced security and user experience in various domains of research and application.

## 3. Materials and Methods

In this section, we explain the dataset used, the preprocessing of the data, the model used, and the parameters of the model.

### 3.1. Sensing Infrastructure Design

**Figure 1:**
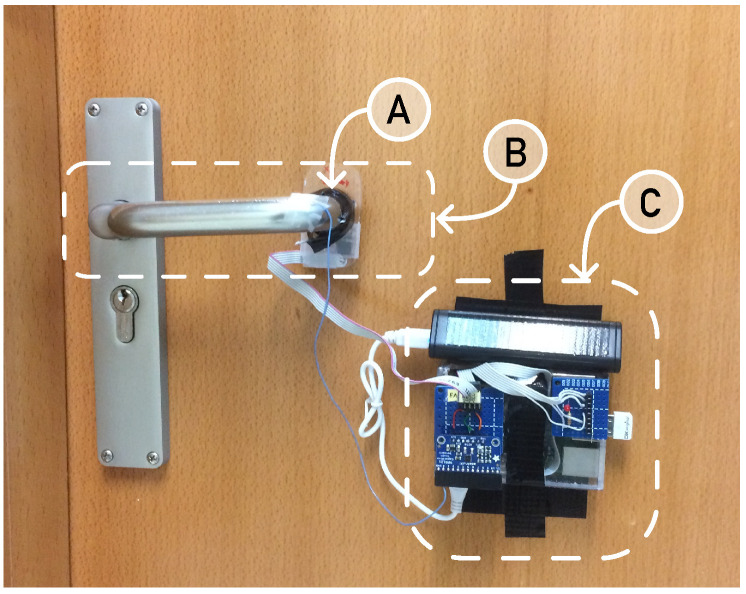
The experimental system is made of a sensorized doorknob (B) with a 9-DOF sensor firmly attached to the handle (A) and controlled by a portable small computer (C). The door is always locked throughout the experiment. The small computer consists of a Raspberry Pi/2+ with a Broadcom BCM2837 ARM7 quad-core processor running at 900 MHz and 1 GB of RAM; it is wired to the sensing element: an MPU 9250 breakboard controlled through an I2C interface programmed in Go on a Jessie Raspbian software stack.

The sensing platform is composed of a 6-DOF sensor breakout board, a microcontroller, and a wireless communication module, as can be seen in Figure 1. More details of the sensing platform can be found in [20]. To summarize, the sensor breakout board is a 6-DOF IMU sensor (MPU-6050) that integrates a 3-axis accelerometer and a 3-axis gyroscope, which are sampled with a frequency of no less than 500 Hz, so some further filtering and subsampling can be made from the original data.

**Figure 2:**
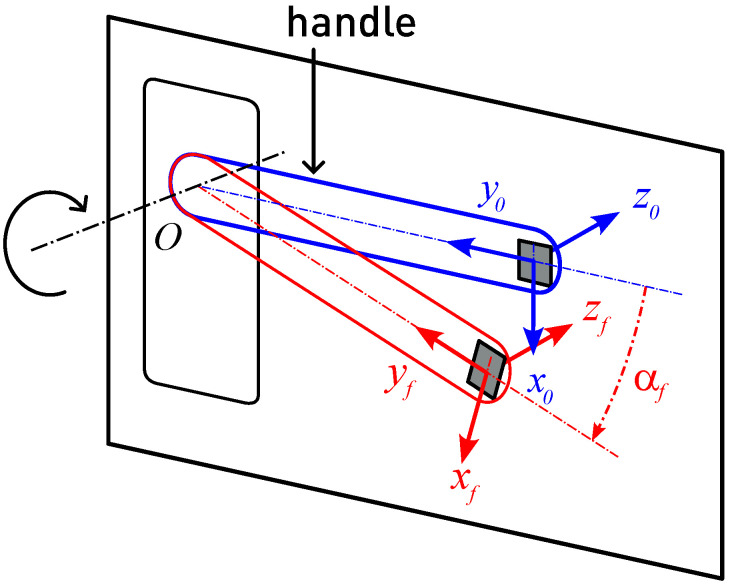
Description of the lever door handle utilized in the experiment. The plate attached to the end of the lever represents the sensor breakout. The sensor’s frame of reference $\left(\vec{x},\vec{y},\vec{z}\right)$ moves together with the lever. At rest, the lever is positioned horizontally, with ${\vec{x}}_{0}$ assumed to point downwards. The $\vec{z}$-axis remains consistently aligned with the rotation axis of the door handle, while $\vec{x}$ and $\vec{y}$ move within a plane that is perpendicular to this rotation axis.

If we neglect the possible mechanical looseness of the door handle and assume that the system is perfectly aligned, the $\omega_{x}$ and $\omega_{y}$ components became 0, and then, $\vec{\omega }\left(t\right)=\left(0,0,\omega_{z}\left(t\right)\right)$. In fact, considering that the vector basis for the sensor system rotates jointly with the door handle, we can consider that ${\dot{\omega }}_{z}\left(t\right)≃a_{x}\left(t\right)-g\cos\theta (t)$ (Figure 2).

**Figure 3:**
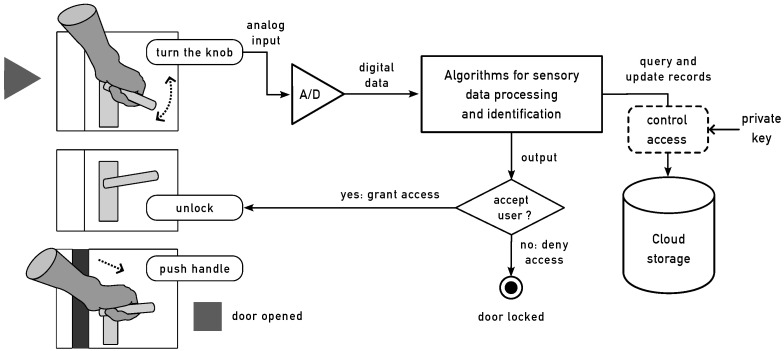
This figure shows a high-level view of our envisioned system. For the sake of illustration, only one is shown, although many more can be deployed as necessary. At the starting point, the user acts on the handle of a locked doorknob. Then, the sensors first perform analog-to-digital conversion of the time-series patterns of movement that a user employs to open the door. The digital signals are then fed into a server that performs the necessary processing for identifying users. The results are stored in a cloud storage device that uses a MySQL database. Since the processed data contain personal information about users, we use a private key so that only authorized personnel can access the database. Eventually, depending on the access permission of the user to unlock the door, the system is capable of feeding a signal to an unlocking system.

Figure 3 shows a high-level view of our envisioned system in which this prototype can be evolved. The expectation is that our doorknob sensor can de deployed at multiple locations simultaneously, such as all the doors in a home or assisted living center. Each doorknob is fitted with our sensor, which first performs analog-to-digital conversion of the patterns of movement that a user employs to open the door. As shown in the sensor design section, the accelerometers obtain temporal data from each user. The digital signals are then fed into a server that performs the necessary processing for identifying users. The results are stored in a cloud storage device that uses a MySQL database. A description of the database and storage is outside the scope of this paper. The reader is referred to our earlier work, which provides details [33]. The processed data contain personal information about users. Hence, we use a private key so that access to the database is restricted and is only permitted for authorized personnel.

#### Dataset

Our dataset comprises a series of vectors derived from angular speeds and accelerations, with time labeled in microseconds. We collected data from a total of 47 individuals (13 female and 34 male), aged between 18 and 68 years. Each participant interacted naturally with a door handle for 20 repetitions on a firmly closed door, resulting in a total of 940 door-opening attempts. None of the participants in the study were affected by any disease or physical condition that would prevent them from performing the act of opening the door.

The first 200 samples of each series capture the system’s rest state before the platform detects a significant change in signal energy. From this moment, the platform collects a fixed number of samples over a duration of 2.5 s, which we determined to be sufficient to gather all necessary data.

Currently, the data are publicly available in the repository of the University of Valladolid [34].

### 3.2. Algorithms for Processing the Sensor Data

We applied the following set of algorithms to process the data obtained from the doorknob sensor. The data were first preprocessed to remove noise. We then trained a machine learning model to identify the user.

**Figure 4:**
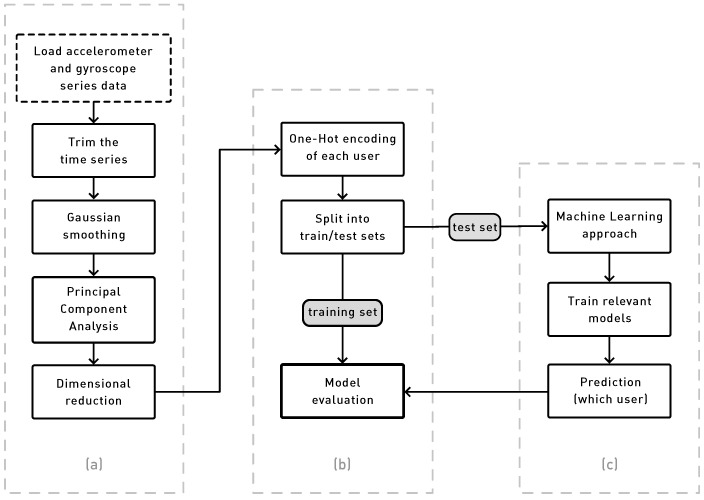
Sensory data processing pipeline. The blocks on the left part (**a**) depict the initial acquisition and data conditioning pipeline tasks that feed data to the training and evaluation platform (**b**). This module is responsible for providing learning patterns to the machine learning system (**c**) and, finally, compares the discriminating capabilities of the whole system.

Figure 4 contains the processing steps applied to the data obtained from the doorknob sensor.

The following steps provide more details about the process.

#### 3.2.1. Data Trimming

**Figure 5:**
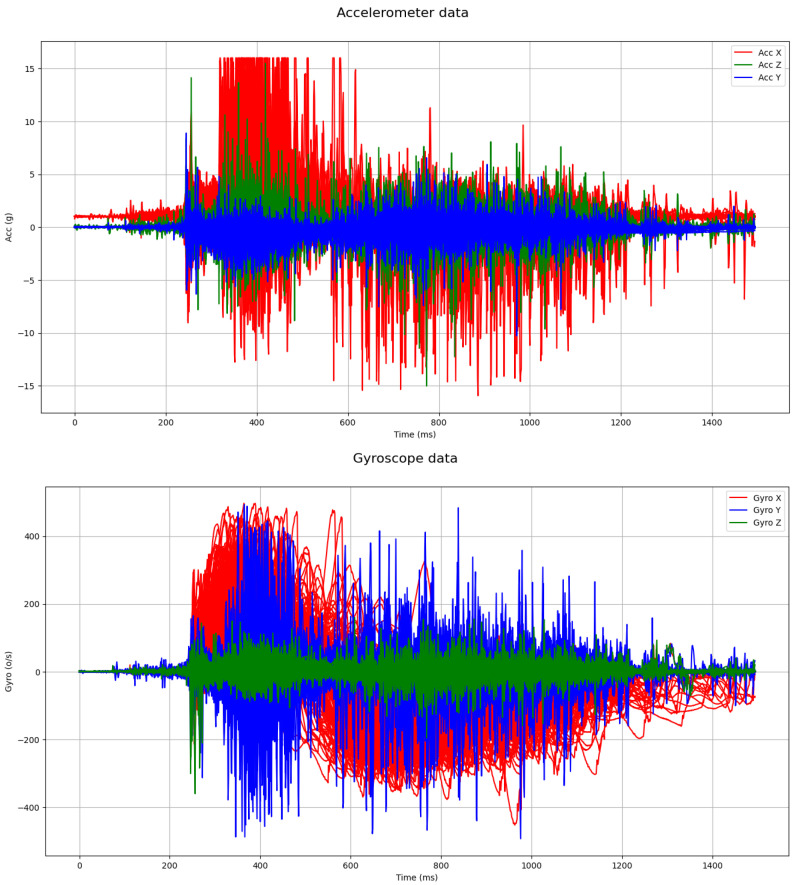
Accelerometer and gyroscope readings across for all users. The x-axis depicts the time in milliseconds, and the y-axis shows the accelerometer (*g*) and gyroscope (°/s) readings on each plot separately. Each dimensional coordinate is represented by a different color based on the axis in order to improve the visibility of the curves.

Figure 5 shows the time-series for the accelerometer and gyroscope readings for all of the users. Each user is encoded with a different color in the plot. We observe that most users finish the operation of turning the doorknob in about one second. Hence, we trim the data so that we only include the samples from 200 to 1400 ms in the plot.

#### 3.2.2. Gaussian Smoothing

**Figure 6:**
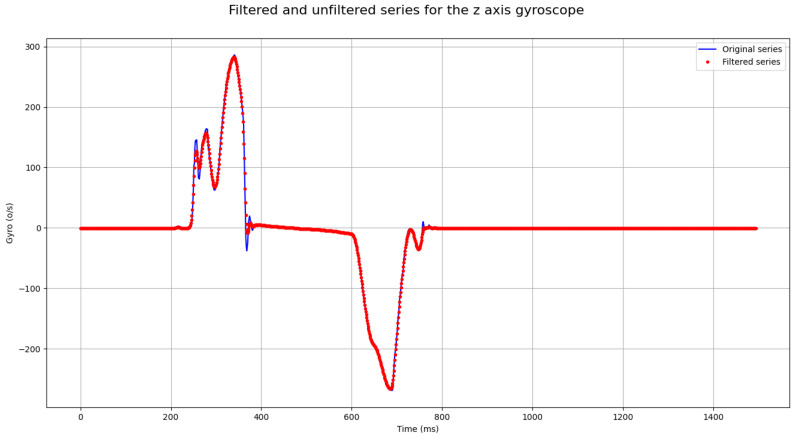
We applied Gaussian smoothing to the signal depicted in Figure 4. This time-series corresponds to the z-axis velocity (°/s) obtained via the z-axis gyroscope along the time in milliseconds. We used a kernel width of 3. The function we utilized was gaussian_filter1d in the Python 3.10.12 open-source library scipy.ndimage [36]. The original signal is shown as the blue line, and the smoothed signal is shown via the red dots to improve the clarity of the two depictions.

Gaussian smoothing is a recommended step for signal processing pipelines, as it combats noise [35]. We applied Gaussian smoothing to the signal, obtaining a slight improvement in the performance of the PCA method. The percentage of variance in the data explained by two dimensions improved from a 99.56% to a 99.8% after applying Gaussian smoothing. Figure 6 shows the result of applying Gaussian smoothing to the time series obtained from the same single individual referred above.

#### 3.2.3. Principal Component Analysis

The next step is to conduct principal component analysis. There are six measurements, or dimensions, for each action to open the doorknob; these are related to the acceleration and gyroscopic motion. Though in principle these measurements can be uncorrelated, we observe in practice that there is strong correlation between these measurements. Hence, principal component analysis is an appropriate technique to reduce the dimensionality of the feature space. We found that using a projection of the original feature space with six dimensions into a new feature space with two dimensions was sufficient to capture the essential variation in the data. This dimensionality reduction will speed up further processing steps in the pipeline and is very desirable.

#### 3.2.4. One-Hot Encoding

The final step in the data processing pipeline is to apply one-hot encoding to the data. This is a standard technique in machine learning to convert categorical data into a form that can be provided to machine learning algorithms. Let N be the number of users. We conducted one-hot encoding of the index i, which varies between 1 and N, where i identifies the user. This helps to establish vectors for training the deep neural network.

#### 3.2.5. Machine Learning Approach

The dataset was split into a training and validation set (80%) and a testing set (20%). The training and validation set was split into training (75%) and validation(25%) sets, respectively. The training set was used to train the model, and the validation was used to tune the hyperparameters of the model. Finally, the testing set was used to evaluate the performance of the model. The model was evaluated using standard accuracy metrics.

We used the Keras framework, which is popular for building deep learning applications [37]. Since layers are the basic building blocks in Keras, we used APIs that addressed each model layer of the neural network.

The model layers comprise the following entities.

- Normalization layer: This layer shifts and scales inputs into a distribution centered around 0 and with a standard deviation of 1.
- LSTM layer: The long short-term memory layer is a recurrent neural network that takes time-series data as input.
- Bidirectional layer: This is a wrapper that is applied to recurrent neural networks to process data in the forward (from past to future) and backward (from future to past) directions.
- Conv1D: Convolutions are the result of performing mathematical operations: mainly, scalar products of the elements of a kernel window of a given size over the input. In this operation, the kernel is a window of a specified size. A filter is a group of kernels, where the number of kernels is specified. This layer produce as many convolutions as the number of filters specified. The goal of this layer is to learn spatial hierarchies of patterns.
- MaxPooling1D: The purpose of this layer is to down-sample the input, reducing its dimensionality. In this operation, the input is divided by a specified integer into discrete sections. Within each, we produce a float value, which is the maximum value within each section.
- GloabalMaxPool1D: We down-sample the input by taking the maximum value over the time dimension.
- Flatten: This flattens the input into shape(none, x), where x is the multiplication of input shapes.
- Dense: This operation utilizes a regular densely connected neural network layer. The neuron in a given layer receives inputs from all neurons in the previous layer. Subsequently, a non-linear transformation is performed to produce the output.
- Dropout: This operation sets randomly selected input units to zero with a specified frequency.

The model stops training if the following two conditions are met:

- The number of training epochs with no improvement in the error is greater than a threshold.
- The training loss is less than 0.1, or the validation accuracy is greater than a threshold.

#### 3.2.6. Model Creation

**Figure 7:**
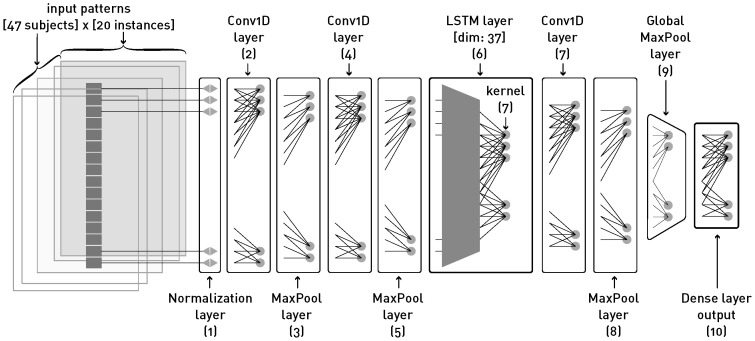
Composition of the deep learning network model: (1) normalization, (2) a one-dimensional convolutional layer (Conv1D), (3) a MaxPooling layer, (4) a one-dimensional convolutional layer (Conv1D), (5) a MaxPooling layer, (6) an LSTM layer, (7) a one-dimensional convolutional layer (Conv1D), (8) a MaxPooling layer, (9) a GlobalMaxPooling layer, and (10) a dense layer on top.

The proposed hybrid model, as depicted in Figure 7, is composed of the following 10 layers: (1) normalization, (2) a one-dimensional convolutional layer (Conv1D), (3) a MaxPooling layer, (4) a one-dimensional convolutional layer (Conv1D), (5) a MaxPooling layer, (6) an LSTM layer, (7) a one-dimensional convolutional layer (Conv1D), (8) a MaxPooling layer, (9) a GlobalMaxPooling layer, and (10) a dense layer on top. The model is trained using the Adam optimizer, which is an extension of stochastic gradient descent. The loss function used is the categorical crossentropy, which is used in multi-class classification problems. The metrics used to evaluate the model are the accuracy and the loss.

The bach size is set to 32, and the number of epochs is set to 20. We used the Adam optimizer, which is a popular optimization algorithm that is an extension of the stochastic gradient descent. Researchers have shown that Adam performs well across different neural network architectures and problem domains [38]. We deployed a categorical crossentropy loss function, which is used in multi-class classification problems. The metrics used to evaluate the model are the accuracy and the loss. To choose the model configuration, we relied on previous research [39], where different configurations of DNNs were investigated, and it was found that an 8-layer network with Adam optimization gave the best performance for prediction problems. In the present work, we used a network of similar size, which also obtained good results.

The hyperparameters of the model are as follows:

- Number of individuals: 47
- Number of epochs: 20
- Batch size: 32
- Optimizer: Adam
- Loss function: Categorical crossentropy
- Metrics: Accuracy and loss
- Neurons in the LSTM layer: 37
- Kernel size: 7

Model creation is carried out through the following steps. Firstly, the sensory data processing pipeline is performed, as shown in Figure 4. Then, the LSTM layers are trained with the data from each user. Finally, classification is performed using the trained model by providing the sensory signals from each user. All the experiments were run in Google Colab.

#### 3.2.7. Model Evaluation

The performance of the resulting model is evaluated through iterative k-fold cross validation, with the value of *k* set to 8. The model is trained on k−1 folds and is tested on the remaining fold. This process is repeated *k* times, with each fold serving as the test set exactly once. The average performance across all folds is used to evaluate the model.

**Figure 8:**
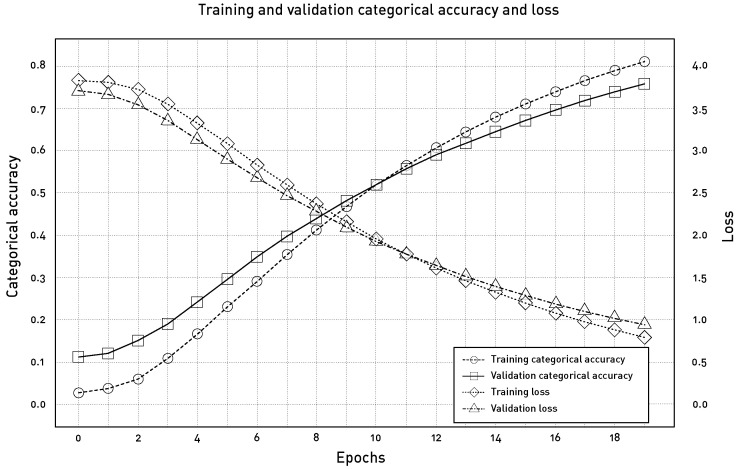
Plot of the training and validation categorical accuracy and loss for every epoch. The left y-axis is valid for the categorical accuracy, while the right y-axis accounts for the loss.

Figure 8 depicts the loss and the categorical accuracy versus the number of epochs for the training and validation datasets.

## 4. Results

We evaluated the performance of the model for identifying users without considering their sex as an input feature. The results are shown in Table 1. The model achieved a general accuracy of 90.2% in identifying users.

**Figure 9:**
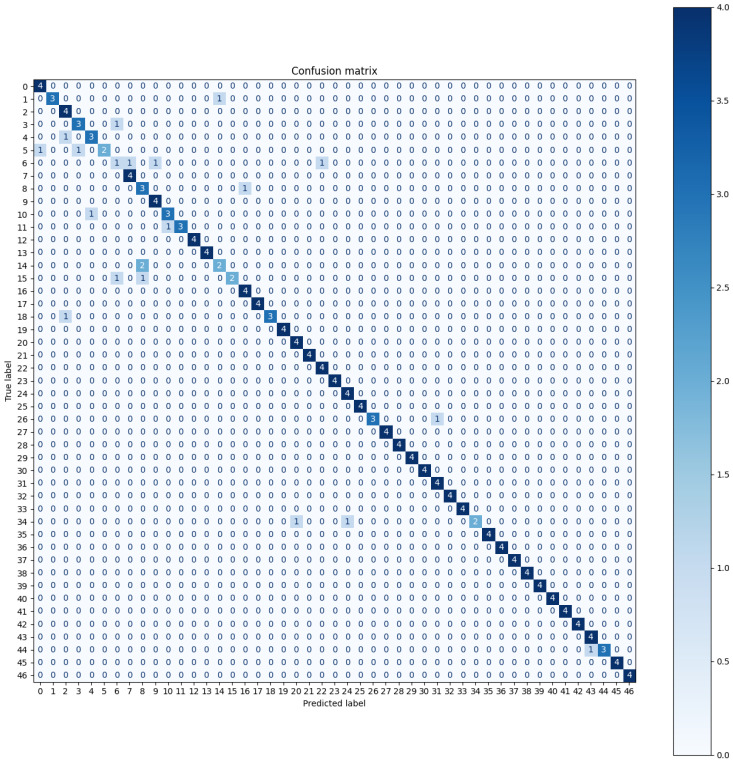
Confusion matrix, where the true label for each user is along the rows, and the predicted label is along the columns.

Figure 9 depicts the confusion matrix, which captures the relationship between the true and predicted labels for the users.

To analyze the results in terms of the confusion matrix, we calculated the true positive rate, true negative rate, false positive rate, and false negative rate for each user. Table 2 summarizes the standard measurements related to classification performance in the evaluation stage, which includes the true positive rate, true negative rate, false positive rate, and false negative rate for each user. As the data of 20 attempts for each user were split into a training and validation set (80%) and an evaluation set (20%), this corresponds to four samples for each user in the evaluation set.

We then investigated the following two questions. What is the influence of sex on the performance of the model? What is the effect of the length of the samples on identification? The second question is relevant as it influences the practical real-time application of the model.

### Influence of Sex and Length of Samples

We analyzed the influence of sex on the identification performance. We separated the samples corresponding to the male and female individuals and calculated the evaluation accuracy following the same procedure described earlier.

In order to analyze the effect of the length of the samples on performance, we examined the following settings: the first 1495 samples, the first 1000 samples, the first 500 samples, and the first 250 samples. Considering that the sampling frequency was about 500 Hz, 1295 samples corresponds to 2.5 s, 1000 samples corresponds to 2 s, 500 samples corresponds to 1 s, and 250 samples corresponds to 0.5 s worth of data.

These two factors were analyzed together, and the obtained results are shown in Table 3.

## 5. Discussion

The results obtained validate the hypothesis that patterns of doorknob manipulation are valid for access control. This expands the set of alternatives to be considered for behavioral biometrics. Our results show that the interaction of a person with the handle of a door when he/she tries to open the door can be used to identify the user with a high degree of accuracy.

As we can see in Table 1, the accuracy of the identification of the users is very high, with an average accuracy of 90.2%. This means that the model is able to correctly identify the user in 90% of cases. This is a very high degree accuracy and shows that the model is able to learn the patterns of user behavior and apply this information to identify the user.

These results suggest that our methodology can be used in a tracking AI system, where the system can track users as they move around the house. This can be used to provide personalized services to a user, such as turning on the lights when the user enters a room or adjusting the temperature when the user enters a house. This can also be used for security purposes, where the system can issue alerts if an unknown person enters the house. In the context of ambient assisting living (AAL), this can be used to monitor the activities of the user and provide assistance when needed.

Putting these results in the context of the works reviewed in the introduction, we can see that our approach is able to achieve higher accuracy than the other approaches. For example, in [21], the accuracy was 89.5%, and in [23] the accuracy was 70.94% when the handle was pressed but the door had not yet moved. Our approach is able to achieve an accuracy of 90.2%, which is higher than other approaches that use a single sensor. Ishida [24] achieved an accuracy of 91.9% by deploying a larger number of sensors than we used.

Furthermore, many of the studies we cited were carried out with much smaller sample sizes. In [21], the experiment was conducted with 12 users, in [23] the experiment was conducted with 4 users, and in [24] the experiment was conducted with 4 users. In contrast, we used a sample size of N = 47 users. This demonstrates the broader validity and the strength of our approach, as it is carried out using a much larger sample size than other researchers have used.

In general, in these types of experiments, it is difficult to compare the results directly since the experimental settings, user behaviors, and sensors are different in each case. As the field advances, it may help to standardize the different sensors and for researchers to share their data. As an example, the field of machine learning has benefited greatly from sharing datasets and organizing competitions [40].

We obtained interesting results by examining the influence of sex on the identification performance. As we can see in Table 3, the accuracy for the identification of female users is higher when only the samples corresponding to female users are considered, with an average accuracy of 97.0%. This represents an improvement of 6.8 percentage points with respect to the general experiment in which the samples corresponded to both male and female users. At the same time, the accuracy of identifying male users decreases slightly by 0.04 percentage points when only samples corresponding to male users are considered, with an average accuracy of 89.8%. This represents a decrease of 0.6 percentage points with respect to the general experiment. These results suggest that the model is sensitive to the sex of the users, with an improvement in the identification of users when male and female users are considered separately. One drawback of this result is that the number of male and female users is not balanced in the dataset, and this could affect the results. We plan to investigate this result further through future research by utilizing a larger sample size with a balanced number of male and female users.

Our investigation of the length of the samples shows that the accuracy of the identification of the users decreases as the length of the samples decreases. The only exception corresponds to the case of male users, where the accuracy of the identification of the users increases slightly when only the first 1000 samples are considered, with an average accuracy of 91.6%. This represents an improvement of 1.4 percentage points with respect to the general experiment in which the samples had a duration of 2.5 s. This is an interesting result, since it shows that the model is able to identify the user even when the samples are shorter. This is important in a real-time applications where the model has to make a decision quickly. The general tendency towards less accuracy is expected, since the model has less information to work with when the samples are shorter. However, the decrease in accuracy is not as large as one might expect, with an average accuracy of 68.5% when only the first 250 samples are considered. This represents a decrease of 21.7 percentage points with respect to the general experiment in which the samples had a duration of 2.5 s.

We can balance the duration of the sample size and the accuracy. From Table 3, for an 80% reduction in the duration of the sample size from 2.5 s to 0.5 s, there is a 31.7% decline in the performance. Based on the specific requirements of the system to be deployed, the implementor can choose the desirable operating point in terms of speed and accuracy.

## 6. Conclusions

We presented a novel approach for user identification in access control systems based on a person’s interaction with a door handle. Our results demonstrate that this method is effective for access control, thereby expanding the range of alternatives for behavioral biometrics. Patterns of interaction with a door handle can be used to identify users with a high degree of accuracy. We deployed deep neural network techniques to achieve this level of accuracy.

We found the model to be sensitive to user sex. Identification accuracy improves when the samples are considered separately for male and female individuals. This is an interesting result that can help drive improvements of such systems in the future.

We determined that the accuracy of the identification is proportional to the length of the samples used in the model, with lower accuracies for smaller sample lengths. The decrease in accuracy is not as large as one might expect, and our results show that the model is able to achieve reasonable performance even when the sample length is decreased. Since the length of samples used is related to the speed of operation, our results are important for real-time applications where the model has to make a decision quickly.

## 7. Future Work

There are several open questions that must be addressed in future work.

One of our pending future issues is how is it possible to extend our model in order to be used in a real-world scenario where a very big dataset and real-time constraints impose further restrictions to the computational platform. This will include performing a broader parameter search for tuning purposes. Although unproven, we are confident that this model could be easily extended to take into account noisy environments and broken time-series. Also, special care must be taken in a security-aware environment where possible malicious readings occur. In this case, our contribution could be a robust input to a multifactorial authentication process.

Regarding the sensitivity of the model to the sex of the users, we plan to investigate this outcome further by utilizing a larger dataset with a balanced number of samples of both sexes.

## Figures and Tables

**Table 1:** K-fold results, including validation and evaluation loss and accuracies.

Average Validation Loss:	0.325
Average Validation Accuracy:	0.912
Average Evaluation Loss:	0.327
Average Evaluation Accuracy:	0.902

**Table 2:** True positive rate (TPR), true negative rate (TNR), false positive rate (FPR), false negative rate (FNR), and accuracy (ACC) over repeated trials for a given user.

User_Id	TPR	TNR	FPR	FNR	ACC
user_00	0.5	0.99	0.01	0.5	0.98
user_01	0.75	1.0	0.0	0.25	0.99
user_02	1.0	1.0	0.0	0.0	1.0
user_03	1.0	0.99	0.01	0.0	0.99
user_04	1.0	0.99	0.01	0.0	0.99
user_05	1.0	1.0	0.0	0.0	1.0
user_06	0.75	0.98	0.02	0.25	0.98
user_07	0.75	0.99	0.01	0.25	0.99
user_08	1.0	1.0	0.0	0.0	1.0
user_09	0.75	0.99	0.01	0.25	0.98
user_10	0.75	0.99	0.01	0.25	0.98
user_11	0.5	1.0	0.0	0.5	0.99
user_12	0.75	1.0	0.0	0.25	0.99
user_13	1.0	1.0	0.0	0.0	1.0
user_14	0.75	0.99	0.01	0.25	0.99
user_15	0.75	0.99	0.01	0.25	0.99
user_16	1.0	0.99	0.01	0.0	0.99
user_17	1.0	1.0	0.0	0.0	1.0
user_18	0.75	1.0	0.0	0.25	0.99
user_19	1.0	1.0	0.0	0.0	1.0
user_20	1.0	1.0	0.0	0.0	1.0
user_21	1.0	0.99	0.01	0.0	0.99
user_22	0.0	1.0	0.0	1.0	0.98
user_23	1.0	1.0	0.0	0.0	1.0
user_24	1.0	1.0	0.0	0.0	1.0
user_25	1.0	1.0	0.0	0.0	1.0
user_26	0.5	1.0	0.0	0.5	0.99
user_27	1.0	1.0	0.0	0.0	1.0
user_28	1.0	0.99	0.01	0.0	0.99
user_29	1.0	1.0	0.0	0.0	1.0
user_30	1.0	1.0	0.0	0.0	1.0
user_31	1.0	1.0	0.0	0.0	1.0
user_32	0.75	1.0	0.0	0.25	0.99
user_33	1.0	1.0	0.0	0.0	1.0
user_34	0.75	1.0	0.0	0.25	0.99
user_35	1.0	0.99	0.01	0.0	0.99
user_36	1.0	0.99	0.01	0.0	0.99
user_37	0.75	1.0	0.0	0.25	0.99
user_38	1.0	1.0	0.0	0.0	1.0
user_39	1.0	0.99	0.01	0.0	0.99
user_40	0.75	0.99	0.01	0.25	0.99
user_41	1.0	1.0	0.0	0.0	1.0
user_42	1.0	1.0	0.0	0.0	1.0
user_43	1.0	1.0	0.0	0.0	1.0
user_44	1.0	0.99	0.01	0.0	0.99
user_45	1.0	1.0	0.0	0.0	1.0
user_46	1.0	1.0	0.0	0.0	1.0

**Table 3:** Accuracy metrics for samples of different lengths, including all users and male-only and female-only groups.

	Identification Accuracy by Sex		
Number of Samples Considered	All Users	Male	Female
First 1295 samples (2.5 s)	**0.902**	0.898	**0.970**
First 1000 samples (2.0 s)	0.896	**0.916**	0.957
First 500 samples (1.0 s)	0.833	0.856	0.903
First 250 samples (0.5 s)	0.685	0.712	0.852

## Data Availability

The data are publicly available in the repository of the University of Valladolid at: https://uvadoc.uva.es/handle/10324/68700.
